# Hepatic and Splenic Hyaloserositis

**DOI:** 10.3390/diagnostics15151949

**Published:** 2025-08-04

**Authors:** Ádám Ferenczi, Karim Rashid, Yaffa Alkawasmi, El Samad Rayan, Sawako Yoshida, Ahmed Friji, Tran Anh Phuong, Tamás Lantos, Anita Sejben

**Affiliations:** 1 Department of Pathology, Albert Szent-Györgyi Medical School, University of Szeged, Állomás utca 1, 6725 Szeged, Hungary; 2 Department of Medical Physics and Informatics, University of Szeged, 6725 Szeged, Hungary

**Keywords:** hyaloserositis, icing sugar spleen, frosted liver, cirrhosis

## Abstract

Hyaloserositis, also known as the icing sugar phenomenon, may be commonly observed during autopsies; however, it is not a well-documented topic with varying nomenclature and etiology, which can be generally defined as an organ being covered with a shiny, fibrous hyaline membrane. In our work, we present the case of a 71-year-old female patient with alcohol-induced liver cirrhosis and subsequent ascites and recurrent peritonitis. During the autopsy, a cirrhotic liver and an enlarged spleen were observed, both exhibiting features consistent with hyaloserositis, accompanied by acute fibrinopurulent peritonitis. Histological examination revealed the classical manifestation of hyaloserositis, further proven by Crossmon staining. The cause of death was concluded as hepatic encephalopathy. During our literature review, a total of seven cases were found. It must be emphasized that no publication describing hyaloserositis from the perspective of a pathologist was discovered. Regarding etiology, abdominal presentations were most commonly caused by serohepatic tuberculosis, while pleural manifestation was observed following trauma. Hyaloserositis may prove to be a diagnostic difficulty in imaging findings, as it can mimic malignancy; therefore, a scientific synthesis is necessary.

**Figure 1:**
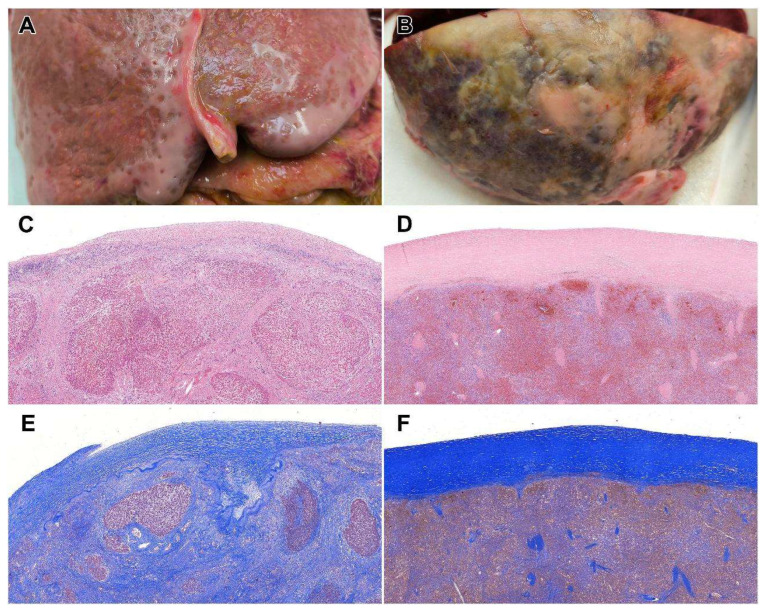
We present the autopsy findings of a 71-year-old female patient with a medical history significant for chronic alcohol dependence, resulting in alcoholic liver cirrhosis, as well as benign essential hypertension, type 2 diabetes mellitus, and systemic atherosclerosis. The patient was initially admitted to the hospital with acute decompensation of liver cirrhosis, accompanied by elevated inflammatory markers that raised clinical suspicion for spontaneous bacterial peritonitis. Despite negative ascitic fluid cultures, empiric antibiotic therapy with ceftriaxone and metronidazole was initiated, leading to a downward trend in inflammatory parameters. During hospitalization, the patient developed mild anemia, which was successfully managed with a transfusion. Ascitic fluid accumulation reached 3000–4000 mL daily, prompting transfer for the placement of a pigtail catheter. At the time of this admission, the patient presented with mild abdominal pain, marked jaundice, and bilateral lower extremity edema. Pigtail catheter placement was successful, and approximately 2000 mL of ascitic fluid was removed daily. Ceftriaxone and metronidazole were continued. Management of edema included intravenous loop diuretics and oral spironolactone. Protein supplementation, both oral and intravenous, was initiated to address hypoalbuminemia. Subsequently, the patient developed diarrhea. Stool cultures were positive for Clostridioides difficile, leading to adjustment of the antibiotic regimen to intravenous metronidazole and oral vancomycin. Despite therapeutic measures, the patient’s clinical condition deteriorated. She experienced recurrent episodes of vomiting followed by loss of consciousness. Ultimately, she succumbed to central cardiorespiratory failure secondary to hepatic encephalopathy. Postmortem examination was requested to clarify the cause of death. Macroscopic evaluation of the abdominal cavity revealed the presence of 900 mL of ascitic fluid. The liver weighed 1210 g and exhibited a nodular, irregular surface with evidence of hyaloserositis. On the cut surface, macroscopic features of cirrhosis were evident (**A**), which were further corroborated histologically by the presence of hyalinized collagen fibers ((**C**): H&E, 2×) and confirmed by Crossmon staining ((**E**): Crossmon, 3×). The spleen was enlarged (330 g) and displayed capsular hyaloserositis ((**B**,**D**): H&E, 2×; (**F**): Crossmon, 3×). Additionally, acute fibrinopurulent peritonitis was observed. Other significant findings included chronic pancreatitis, marked systemic atherosclerosis, and passive congestion of multiple internal organs. The cause of death was determined to be hepatic encephalopathy. The underlying condition was chronic alcohol abuse, leading to liver cirrhosis, portal hypertension, and ultimately, terminal hepatic parenchymal and vascular decompensation. Comorbidities included benign essential hypertension—contributing to hypertensive heart and renal disease—as well as type 2 diabetes mellitus. The fibrinopurulent peritonitis may have resulted from bacterial transmigration. Splenomegaly was likely secondary to portal hypertension and systemic congestion. Although hepatic pathologies such as congestion, cirrhosis, and hepatitis are frequently encountered during autopsy, hyaloserositis remains an uncommon finding. To date, only seven cases involving this phenomenon in various organs have been reported in the literature. Notably, one of these references serves primarily as a summary of imaging characteristics rather than a formal case report [1,2,3,4,5,6,7,8,9,10]. Affected organs include the liver (*n* = 4), pleura (*n* = 3), and spleen (*n* = 1). The mean age of affected patients was 46.6 years (range: 17–72), with a male-to-female ratio of 6:1 [1,2,3,4,5,6,7]. A comprehensive summary of these cases is presented in Supplementary Table S1. While all previously published cases include photographic documentation, our report is the first to incorporate a formal literature review. In terms of etiology, hepatic hyaloserositis has thus far only been documented in association with hepatic tuberculosis. Based on current findings, this condition should be considered a rare serohepatic manifestation. Several authors have described the so-called “frosted liver” appearance on imaging, characterized by subcapsular necrotic lesions [1,2,3,4]. Israrahmed et al. further underscore the diagnostic value of imaging in serohepatic tuberculosis, advocating for its inclusion in the differential diagnosis, particularly in endemic regions where subcapsular hepatic lesions exhibit a “sugar-coating” or “icing sugar” appearance. However, all imaging-focused publications consistently highlight the diagnostic challenges posed by isolated hepatic tuberculosis. These challenges stem from the condition’s nonspecific clinical manifestations, its frequent omission from initial differential diagnoses, and its radiological overlap with other hepatic pathologies [1,2,3,4]. Clinically, the most common presentation associated with abdominal hyaloserositis—typically in the context of serohepatic tuberculosis—included mild abdominal pain (*n* = 3), fever (*n* = 1), weight loss (*n* = 1), and hematemesis (*n* = 1) [1,3,4]. Radiologically, ultrasound imaging revealed subcapsular hypoechogenic lesions in three cases and hepatomegaly in one. Contrast-enhanced computed tomography (CT) further delineated these lesions, revealing hypoenhancing hypodense areas in two cases and mild hyperenhancement in another, along with consistent capsular thickening across both imaging modalities. One case also presented with centrally necrotic mesenteric and iliac lymphadenopathy on contrast-enhanced CT. Notably, in the case where high-resolution chest CT was performed, no pulmonary signs of tuberculosis were identified [1,3,4]. Histopathological findings described in the literature include epithelioid cell granulomas, caseous necrosis, purulent exudates, and the presence of acid-fast bacilli in fine-needle aspiration samples. In one case, lymph node biopsy demonstrated Langhans-type giant cells, epithelioid cells, and a lymphoplasmacytic infiltrate [1,3,4]. In contrast, Brønserud et al. reported a pleural hyaloserositis case discovered intraoperatively, proposing a possible link to prior thoracic trauma or chronic inflammation rather than tuberculosis [5]. Similarly, Galatius-Jensen et al. emphasized the diagnostic complexity of pleural hyaloserositis, noting that its radiological appearance can mimic malignancy. Histopathological examination in their cases revealed lymphocytic infiltrates, again supporting an association with chronic inflammation [7]. Clinical manifestations in pleural cases included fatigue (*n* = 1), fever (*n* = 1), and chest pain (*n* = 1). Imaging data on pleural hyaloserositis remain limited, with Galatius-Jensen et al. describing well-circumscribed pleural lesions identified via tomography [7]. While no autopsy findings were available, one case described a small hyaloserositic lesion on the visceral pleura observed during thoracic surgery [5]. Histologically, pleural hyaloserositis was characterized by thickened, partially hyalinized connective tissue with sparse lymphocytic infiltration—again implicating chronic inflammation as a potential aetiological factor [7]. Swami et al. reported a case of hyaline perisplenitis, or “icing sugar spleen,” identified incidentally during autopsy. Macroscopically, the spleen displayed multiple white nodules and plaques (2 mm to 1 cm in diameter) on the capsule, with no parenchymal involvement. Histological examination confirmed hyalinized collagen deposition confined to the splenic capsule. Unfortunately, further clinical details were not provided in the case description [6]. In the present case, recurring episodes of peritonitis may have contributed to the development of hyaloserositis in both the liver and spleen. Importantly, there was no clinical or pathological evidence of Mycobacterium tuberculosis infection, thereby allowing examination of this morphological entity outside the context of serohepatic tuberculosis, which remains the most frequently cited etiology in the literature. Given the inconsistent terminology and fragmented data regarding the etiology, imaging characteristics, and differential diagnosis of hyaloserositis, there is a clear need for a systematic classification of this phenomenon. While often considered rare, its actual prevalence may be underestimated, especially in autopsy series. This underscores the importance of further investigation and standardization in both clinical and pathological contexts.

## Data Availability

The original contributions presented in this study are included in the article. Further inquiries can be directed to the corresponding author.

## Supplementary Materials

The following supporting information can be downloaded at: https://www.mdpi.com/article/10.3390/diagnostics15151949/s1, Table S1: Results of the literature review [1,2,3,4,5,6,7].

## Abbreviations

The following abbreviations are used in this manuscript:

CT	Computed tomography
FNA	Fine needle aspiration
HE	Hematoxylin and eosin
HRCT	High-resolution computed tomography
NA	Not applicable
US	Ultrasound
