# Supplementary material for: Hepatic and Splenic Hyaloserositis
Source: Diagnostics (Basel). 2025 Aug 4;15(15):1949. doi: 10.3390/diagnostics15151949 (PMC12346530; doi:10.3390/diagnostics15151949)
Supplement: Supplementary file 1 [file diagnostics-15-01949-s001.zip › diagnostics-3768280-supplementary.pdf]

**Author and year of publication**	**Nomenclature**	**Affected organ**	**Case number (n)**	**Gender (m=male, f=female)**	**Age (years)**	**Clinical presentation**	**Imaging results**	**Autopsy results**	**Microscopic signs**	**Follow-up data (months)**
Galatius-Jensen et al., 1965 (7)	Hyaloserositis	Pleura	2	Male	46; 49	Tiredness; short episodes of fever	Tomography: 17 x 14 mm well circumscribed density; 20 x 10 mm density	NA	Surgical resection: Connective tissue proliferation, partly hyalinised with few lymphocytes; thickened pleura with fibrotic, hyalinised changes and few lymphocytes	NA
Jain et al., 2016 (1)	Frosted	Liver	1	Female	24	Serohepatic tuberculosis with non-specific abdominal pain hematemesis	US: hepatomegaly, peritoneal thickening, subcapsular hypoechogenic lesions; Contrast	NA	FNA: epithelioid cells and caseous necrosis	6

							CT: hypodense hypoenhancing subcapsular lesions			
Indiran et al., 2017 (2)	Frosted / sugar-coated	Liver	NA	NA	NA	Serohepatic tuberculosis	NA	NA	NA	NA
Swami et al., 2018 (6)	Icing sugar / perisplenitis	Spleen	1	Male	65	NA	NA	Incidental finding of white nodules and plaques 2 mm -1 cm in size on the enlarged spleen (10 x 7.5 x 5 cm), no involvement of splenic parenchyma	Post-mortem histology: Hyalinised collagen fibers over the capsule	NA
Brønserud et al., 2020 (5)	Hyaloserositis	Pleura	1	Male	72	Chest pain	NA	Small "heart-shaped" area of hyaloserositis on visceral	-	NA

								pleura detected during surgery		
Israrahmed et al., 2021 (3)	Frosted / sugar-coated	Liver	1	Male	53	Serohepatic tuberculosis with vague abdominal pain	US: subcapsular hypoechogenic lesions; Contrast CT: multiple hypodense, hypoenhancing nodular subcapsular lesions, thickened capsule, multiple mesenteric and iliac lymph nodes with central necrosis	-	Lymph node biopsy: Langhans type giant cells, epithelioid cells, lymphoplasmacytic infiltrate	3
Dahal et al., 2024 (4)	Frosted / sugar-coated	Liver	1	Male	17	Serohepatic tuberculosis with vague abdominal discomfort and pain, fever, weight loss	US: subcapsular hypoechogenic lesions, central liquefaction; Contrast CT: small hypodense lesion, mild hyperenhancemen	-	FNA: purulent inflammation and acid-fast bacilli	1

							t and thickening of the liver capsule;<br>Chest HRCT: no signs of tuberculosis			
Our case	NA	Liver and spleen	1	Female	71	Mild abdominal pain, pronounced jaundice and swollen legs	NA	Mildly shrunken liver due to cirrhosis, and hyaloserositis, congested spleen with hyaloserositis	Post-mortem histology: collagen deposition on the hepatic and splenic surface	NA

Supplementary Table S1 - Results of the literature review

Abbreviations: CT - Computed tomography, FNA - Fine needle aspiration, HRCT - High-resolution computed tomography, NA - Not applicable, US - Ultrasound
